# Feasibility of a Noninvasive Operability Assessment in Chronic Thromboembolic Pulmonary Hypertension under Real-World Practice

**DOI:** 10.3390/diagnostics10100855

**Published:** 2020-10-21

**Authors:** Adriana Rodriguez Chaverri, Yolanda Revilla Ostolaza, Maria Jesus Lopez-Gude, María Teresa Velazquez, Ines Ponz de Antonio, Sergio Alonso Charterina, Agustin Albarran Gonzalez-Trevilla, Marta Perez Nunez, Jose Luis Perez Vela, Rafael Morales Ruiz, Juan F. Delgado Jimenez, Fernando Arribas Ynsaurriaga, Jose Maria Cortina, Pilar Escribano Subias

**Affiliations:** 1 Hospital Universitario Doce de Octubre, 28041 Madrid, Spain; adrianarodriguezchaverri@gmail.com (A.R.C.); yolanda.revilla@salud.madrid.org (Y.R.O.); mariajesus.lopez@salud.madrid.org (M.J.L.-G.); maitevel05@gmail.com (M.T.V.); ines.ponz@gmail.com (I.P.d.A.); sacharterina@salud.madrid.org (S.A.C.); agustin.albarrang@hotmail.com (A.A.G.-T.); martapereznu@gmail.com (M.P.N.); perezvela@yahoo.es (J.L.P.V.); rmoralesruiz@yahoo.es (R.M.R.); juan.delgado@salud.madrid.org (J.F.D.J.); fernando.arribas@salud.madrid.org (F.A.Y.); josemaria.cortina@salud.madrid.org (J.M.C.); 2 Fundación para la Investigación Biomédica del Hospital Universitario 12 de Octubre (FIBH12O), 28041 Madrid, Spain; 3 CIBER de Enfermedades Cardiovasculares (CIBERCV), 28029 Madrid, Spain; 4 ERN-Lung-Pulmonary Hypertension; 5 Centro de Referencia Nacional de Hipertensión Pulmonar Compleja, Spain

**Keywords:** hypertension, pulmonary, pulmonary embolism, endarterectomy

## Abstract

This study aimed to evaluate the feasibility of a noninvasive operability assessment of chronic thromboembolic pulmonary hypertension (CTEPH) based on multidetector computed tomographic angiography (MCTA). Up to 176 patients were evaluated from January 2016 to April 2018. Throughout the first phase, the initial surgical decision was made based on MCTA with further analysis of pulmonary angiography (PA) in order to evaluate in which cases the initial decision was not modified by PA. During the second phase, PA was limited to patients judged inoperable based on MCTA or those whose assessment was not possible. Patients deemed operable (50%) based on MCTA along the first phase had been adequately classified, as PA did not modify the initial decision in all but one patient. Comparable results were obtained throughout the implementation phase. Regarding operated patients, the decision of operability was based solely on MCTA in 94% of those with level I disease, in 75% with level II, and 54% with level III. This approach enabled shorter periods of time to complete surgical assessment and the avoidance of PA-related morbidity. Baseline parameters, postoperative measures, and survival rates at 1 year after surgery were comparable in both phases. Noninvasive operability assessment is feasible in a subset of CTEPH patients and optimizes surgical candidacy evaluation.

## 1. Introduction

Chronic thromboembolic pulmonary hypertension (CTEPH) represents the third most common cause of pulmonary hypertension (PH) [1] and the only one that is potentially curable [2]. The diagnosis of CTEPH requires the demonstration of pulmonary hypertension on right heart catheterization, along with mismatched perfusion defects on ventilation/perfusion (V/Q) scintigraphy and signs of CTEPH in advanced imaging techniques [3].

Once the diagnosis is confirmed, the next pivotal step consists of assessing the suitability for pulmonary endarterectomy (PEA), which offers the best chance of improved long-term outcomes [4,5]. The operability assessment of patients with CTEPH is complex and accounts for surgical accessibility of the thrombi, concordance between surgically accessible vascular obstruction and pulmonary vascular resistance, and evaluation of underlying comorbidities prohibiting PEA [3,4].

The advanced imaging techniques recommended for the operability assessment of CTEPH include multidetector computed tomographic angiography (MCTA), magnetic resonance (MR) imaging, and conventional pulmonary angiography (PA) [3].

PA is still considered the gold standard for the assessment of pulmonary vasculature. Nevertheless, its routine use is being challenged by advances in noninvasive technology such as MCTA, which has proven high sensitivity and specificity in detecting thromboembolic disease at lobar and segmental levels [6,7]. Additionally, although PA is considered a safe technique, it is an invasive procedure with potential complications [8,9].

The work in recent years have resulted in an increasing CTEPH imaging expertise at our institution alongside a growing healthcare burden as a national CTEPH referral center [10]. We have therefore sought to evaluate the performance of a noninvasive operability assessment in patients with CTEPH as a strategy to optimize surgical candidacy appraisal in relation to quality of care, efficiency, cost reduction, and patient safety.

## 2. Materials and Methods

### 2.1. Study Protocol

As a national CTEPH referral center, we assessed patients with presumed CTEPH, either diagnosed at our institution or oftentimes referred from external hospitals, at a weekly multidisciplinary team (MDT) meeting.

**Figure 1:**
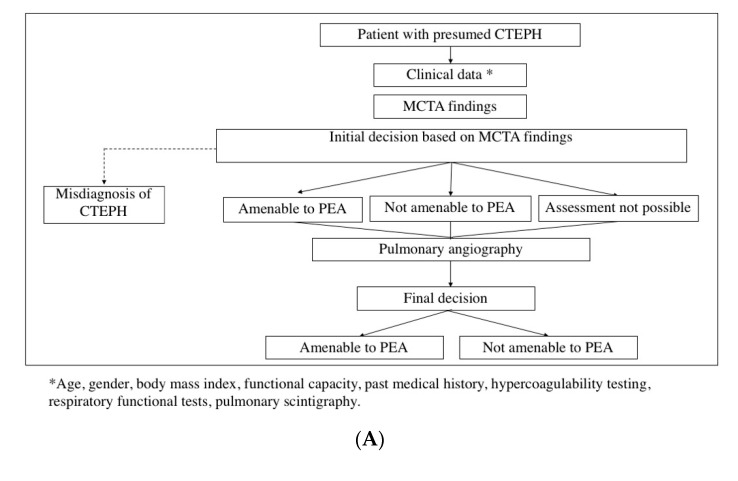

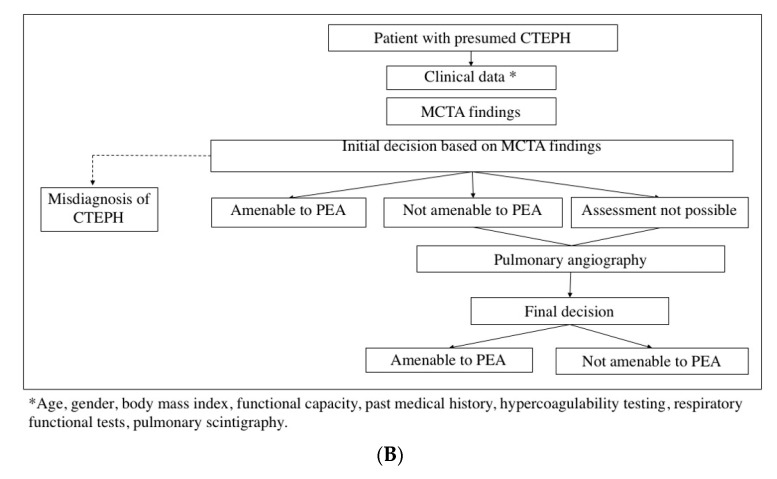
Patient flow assessment performed in the first pilot phase. Every patient was assessed with both multidetector computed tomographic angiography (MCTA) and pulmonary angiography (PA) at the multidisciplinary team (MDT) meeting (**A**). Patient flow assessment during the second “implementation” phase. PA was restricted to patients deemed inoperable based on CT or patients for whom assessment was not possible (**B**). CTEPH = chronic thromboembolic pulmonary hypertension; MCTA = multidetector computed tomographic angiography; PEA = pulmonary endarterectomy.

Upon feasibility analysis of noninvasive operability assessment, the study was divided into two consecutive phases. In the pilot phase, which was developed from January 2016 to March 2017, every patient was assessed at the MDT meeting with both MCTA and PA: MCTA findings were initially analyzed, ultimately deciding which patient was either (1) suitable for surgery, (2) unsuitable for surgery, or (3) whose assessment was unattainable (either because of suboptimal MCTA imaging or due to thromboembolic disease at the limit of surgical accessibility for PEA). Once classified in one of these three groups, PA images were evaluated in order to analyze in which patients the initial decision of operability based on MCTA imaging was unmodified by PA findings (Figure 1A).

During the second phase, implemented from April 2017 to April 2018, the non-invasive operability assessment was launched. In this period, MCTA was performed as the only imaging technique prior to the MDT meeting. A complementary PA was only conducted in two patient groups: (1) those whose assessment was unachievable (due to either suboptimal MCTA imaging or thromboembolic disease at the limit of surgical accessibility); (2) patients judged inoperable based on MCTA, in order to ensure that no operable patient was misclassified as inoperable and denied PEA (Figure 1B).

### 2.2. CTEPH Diagnosis and Image Analysis

Every patient assessed at our MDT meeting had been presumably diagnosed with CTEPH according to current CTEPH guidelines [3]. Nonetheless, differential diagnosis with alternative conditions was sometimes necessary for certain patients that presented with features mimicking CTEPH.

Our institution performed MCTA on a Philips Brilliance 64-slice computed tomographic (CT) scanner. Proximal disease was defined as lesions in the proximal main, lobar, and proximal segmental arteries. Mid and distal segmental and subsegmental branches were considered peripheral disease.

An overall image quality assessment was performed for each MCTA study. Image quality rating was determined by an optimal intravascular enhancement of vascular structures of more than 120 UH, a proper assessment of the whole pulmonary vasculature up to subsegmental branches with good signal-to-noise ratio, absence of motion artifacts, and a slice thickness of 1 mm. Note that this study was conducted in a real-world practice setting with numerous externally referred CT exams. Therefore, optimal CT image quality could not be ensured for all of them.

As a national CTEPH referral center, at least 100 CTEPH cases were annually evaluated over the last 4 years at our center. All of the MCTA studies are analyzed by thoracic imaging unit radiologists, specialized in CTEPH assessment (minimum of 4 years’ experience).

### 2.3. Intraoperative CTEPH Classification and Hemodynamic Reassessment after PEA

Intraoperative classification of the thromboembolic disease described by the University of California, San Diego group [11] was applied to every operated patient. Additionally, this served as an internal validation tool for our proposed noninvasive assessment of operability. A systematic invasive hemodynamic evaluation was performed in all the patients at 1 year after PEA.

### 2.4. Statistical Analysis

All data were expressed as mean and standard deviation (normal distribution) and as median with range (absence of normal distribution). Changes from baseline were evaluated with a paired t-test (continuous variables) and with χ^2^ test (ordinal variables). Survival at 1 year after PEA and factors predicting outcome were analyzed using Cox proportional hazard regression. Significance was determined at *p* < 0.01.

Intra and interobserver variability were evaluated on a sample of 20% of optimal MCTA studies, regarding the level of agreement on the need to perform a complementary PA. For the intraobserver variability evaluation, 10 MCTA studies were selected and blindly reanalyzed with a 4–6 months lapse from the prior analysis. For the interobserver variability evaluation, performed by two radiologists with the highest (10 years) and the lowest (4 years) experience, 40 cases were individually and blindly evaluated.

### 2.5. Ethical Statement

Written informed consent was obtained from all participants in this study, who agreed to provide their data to the Spanish Registry of Pulmonary Hypertension. The Spanish Registry of Pulmonary Hypertension was nationally approved in 2008 by Hospital Universitario de Cruces and afterwards, due to European regulation changes, by our institutional review board (Instituto de Investigacion Hospital Doce de Octubre) on the 20th April 2016. The project identification code assigned was 16/102.

## 3. Results

A total of 192 patients were prospectively evaluated from January 2016 to April 2018 at the MDT meeting: 89 patients in the first phase and 103 patients in the second phase.

Among the whole patient series, 16 patients were excluded from further analysis: 9 of them because of thromboembolic disease with no PH, and the remaining 7 patients because of final diagnosis of PH secondary to alternative causes different from CTEPH, in which the misdiagnosis of CTEPH had been based on V/Q lung perfusion defects. Final diagnosis among the latter were pulmonary veno-occlusive disease (PVOD) (4 patients), idiopathic PAH (1 patient), HIV-related PAH (1 patient), and group 3 PH secondary to pneumoconiosis (1 patient).

Regarding patients with final diagnosis of PVOD, one of them presented with disproportionately severe PH with an isolated subsegmental perfusion impairment with severely reduced diffusion lung capacity for carbon monoxide (DLCO). MCTA showed suggestive PVOD findings, and PA helped to definitely rule out CTEPH. Genetic testing was positive for a biallelic mutation in EIF2 AK4, confirming PVOD. In the remaining three patients with a final diagnosis of PVOD, suspicion of CTEPH had been drawn on mismatched perfusion defects in V/Q scintigraphy, with no CTEPH signs in advanced imaging. The patient with an HIV-related PAH form presented with an isolated segmental defect on pulmonary scintigraphy along with in situ thrombosis and extensive calcification at various levels in the pulmonary vascular bed. The patient with a definitive diagnosis of idiopathic PAH had mismatched perfusion impairment on V/Q scintigraphy and no CTEPH signs in additional studies. Lastly, the final diagnosis was group 3 PH secondary to pneumoconiosis in one patient, an occupational lung disease caused by the inhalation of dust in mines, who also presented with impaired perfusion at V/Q lung scan. PA was performed in all of these patients to adequately rule out CTEPH.

The average age of the cohort with a definitive diagnosis of CTEPH was 59.1 (±15 years). A total of 60% were women, with a mean pulmonary arterial pressure (mPAP) of 47 mmHg (±16 mmHg); 49% were in WHO functional class II and 48% in WHO functional class III.

**Figure 2:**
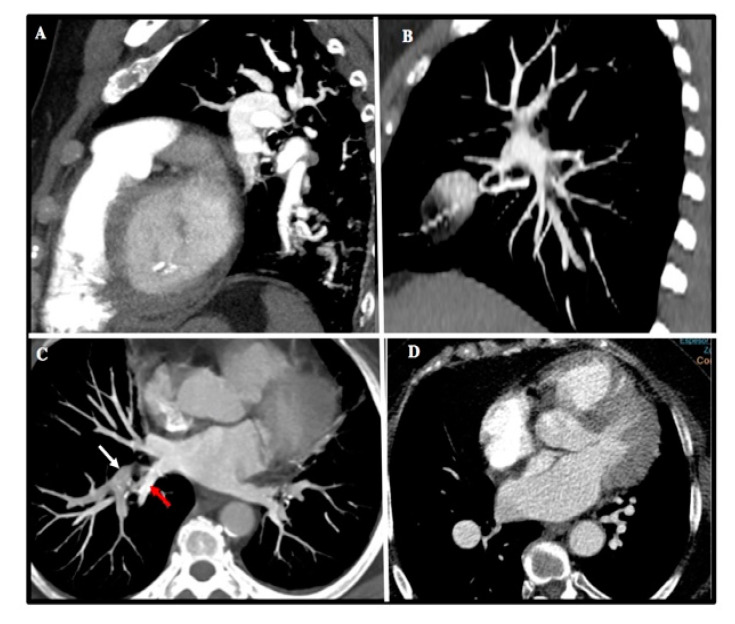
Common imagen artifacts found in MCTA. (**A**) Motion artifact at lower lobes. (**B**) CT stair artifact due to inadequate slice thickness. (**C**) Suboptimal contrast opacification of right upper lobe artery (white arrow) and inferior right vein (red arrow). (**D**) CT image noise in an obese patient.

Up to 89% of MCTA studies were considered optimal. The most frequent causes of suboptimal studies were slice thickness greater than 3 mm (38%), poor opacification (25%), respiratory motion artifact (11%), obesity (9%), and improper breath-holding (9%) (Figure 2).

When there was the need to perform a complementary PA based on MCTA findings, we found excellent intra and interobserver agreement, with a square-weighted Kappa of 0.8 and 0.85 respectively.

### 3.1. Decision Flowchart at MDT Meeting

**Figure 3:**
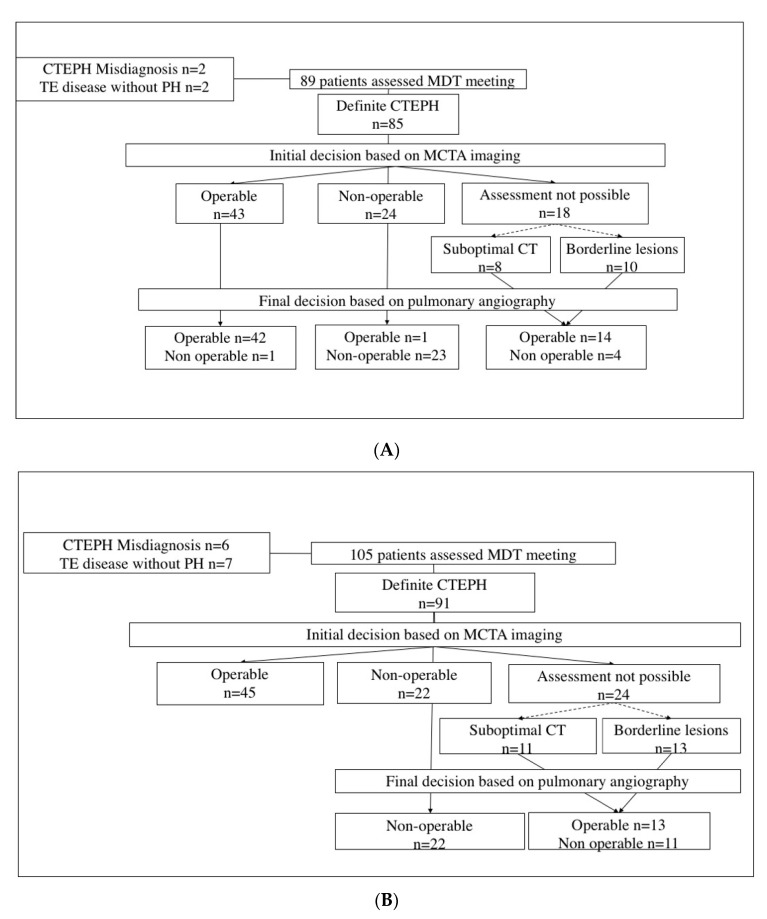
Decision flowchart in the first phase (**A**). Decision flowchart in the implementation phase (**B**). CTEPH = chronic thromboembolic pulmonary hypertension; TE= thromboembolic; PH= pulmonary hypertension; MDT = multidisciplinary team; MCTA = multidetector computed tomographic angiography; CT = computed tomography.

During the first phase, 50.6% of patients were initially deemed suitable for PEA based on MCTA imaging. Among them, the initial decision of operability was modified by PA only in one patient, where the absence of proximal disease at the PA compared to MCTA findings was attributed to a long time lapse between MCTA and PA performance. On the other hand, among patients judged inoperable based on MCTA imaging (28.2%), one patient was relocated as suitable for PEA after performing PA because of unobserved proximal disease on MCTA (Figure 3A).

In the second phase, the operability decision was solely based on MCTA imaging in 49.4% of patients, as they were overtly operable. In contrast, the remaining 50.6% of patients needed a PA to make the final operability decision: in 26.4% of them, surgical assessment based on MCTA had not been possible (either because of suboptimal MCTA imaging or non-overt surgically amenable lesions on MCTA imaging), and 24.2% of them had been judged inoperable based on MCTA (Figure 3B).

### 3.2. Final Treatment, Intraoperative Classification, and Postoperative Outcome

**Figure 4:**
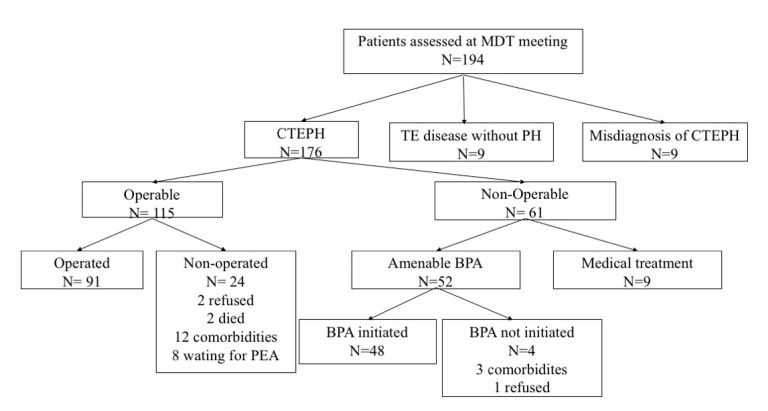
Patients’ disposition according to final treatment.

Patients’ disposition according to the final treatment is summarized in Figure 4.

**Figure 5:**
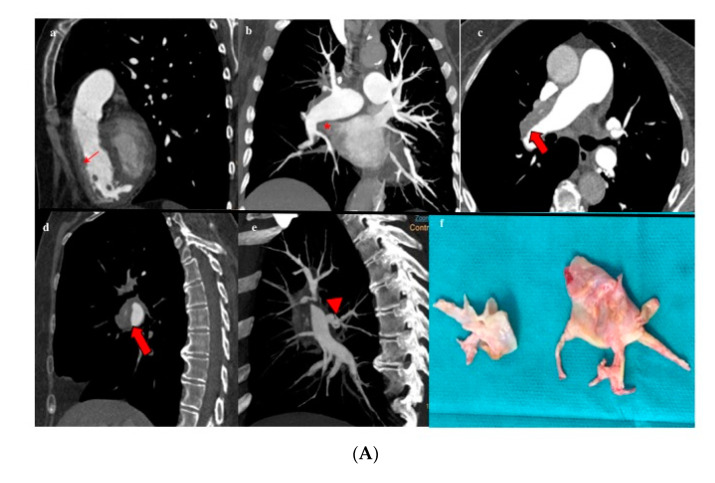

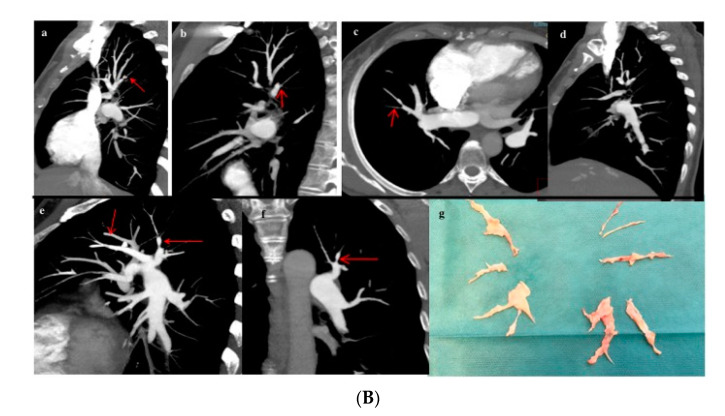
Patient with proximal CTEPH (**A**). (**a**,**b**) Rectified interventricular septum (red thin arrow) and reduction of venous return (*). (**c**–**e**) Thromboembolic disease affecting the main pulmonary artery (red thick arrow) with extension to the right upper lobar artery (arrowhead). (**f**) Surgical specimen showing level I disease. Patient with distal CTEPH (**B**). (**a**–**f**) Organized intravascular material (thin red arrows) is identified at distal-segmental/subsegmental levels in all the pulmonary lobes. (**g**) Surgical specimen showing level III disease.

Amidst those patients who underwent PEA (*n* = 91), 18.7% had level I disease (Figure 5A), 45.1% level II disease, and 36.3% level III disease (Figure 5B). Table 1 summarizes the proportion of intervened patients for whom the surgical decision was based on MCTA compared with those who needed a complementary PA regarding intraoperative CTEPH classification. In up to 54% of intervened patients with level III disease, a decision on operability had been made exclusively on MCTA.

Survival rates at 1 year after PEA in both initial and implementation phases of 93.7 % and 95.4%, respectively, were comparable. Hemodynamic parameters at invasive reassessment at 1 year after surgery in both groups are shown in Table 2.

### 3.3. Efficiency Analysis and Safety Concerns

Shorter time periods to fulfill initial clinical assessment prior to the MDT meeting were achieved in the implementation phase compared with those initially obtained (median waiting times of 110 days; IQR 32–172 vs. 130 days; IQR 59–278; *p* < 0.01).

Among the 131 patients with CTEPH who underwent pulmonary angiography, minor complications occurred in 3 patients (2.3%): femoral hematoma that did not require transfusion (1 patient), allergic reaction to iodine contrast with prompt response to medication (1 patient) and bleeding at the femoral access without transfusion requirements (1 patient). No deaths or major complications related to the technique occurred.

## 4. Discussion

To the best of our knowledge, this is the first study showing that a noninvasive assessment of operability is feasible in a substantial proportion of patients diagnosed with CTEPH. The development of an optimized care process was deemed essential for our institution, a national CTEPH referral center with up to 90% of external patients, in order to make the surgical candidacy assessment for PEA more efficient in terms of shortened waiting periods, reduced costs and patient displacements, as well as increased patient safety.

We obtained surgical rates of 65%, which were comparable with those previously reported in large series such as the large-scale, international registry of patients with CTEPH with surgical rates of 63% [12]. Additionally, our approach did not negatively impact on surgical outcomes in terms of perioperative mortality and hemodynamic improvement at reassessment. Residual PH rate after PEA, which is widely established as a significant prognostic factor in terms of survival [13], not only did not differ significantly between the two phases but tended to be lower in the implementation phase.

Our study aimed to deploy a novel surgical assessment for patients with CTEPH. With this scope, we observed that in the pilot phase, up to 50% of the patients who were deemed operable based on MCTA findings had been adequately classified as such, as PA did not modify the initial decision of operability in all but one of them (where the discrepancy between MCTA and PA findings were justified by a significant time lapse between both procedures). Additionally, comparable results were obtained in the implementation phase, in which surgical suitability was decided solely on MCTA in half of the patients. In the remaining patients, PA was still necessary to make a final operability decision ensuring that no operable patient was misclassified.

Our results indicate that PA was needed in up to 10.8% of the patients to adequately assess surgical candidacy because of suboptimal CT imaging. The proportion of optimal MCTA studies may be foreseeably improved by greater radiological expertise gained in external referral centers alongside with new-generation CT scanner equipment and improved examination protocols [14]. Moreover, CT will presumably gain popularity as a noninvasive hemodynamic monitoring tool in CTEPH, as several CT scores have shown a high correlation with hemodynamic parameters and surgical success rates [15].

It should be highlighted that CT imaging helps to evaluate not only vascular abnormalities that may suggest CTEPH but also alternative parenchymal or vascular disorders that may present with V/Q lung scanning defects. High incidence rates of positive V/Q studies (43.1%) have been reported in recent series among patients with no thromboembolic disease [16]. In our series, 4% of the patients referred as CTEPH were based on impaired perfusion defects on V/Q scanning and displayed no thromboembolic disease in MCTA nor in PA.

In order to perform an internal validation of the proposed operability assessment, we sought to complete the performance analysis by determining the proportion of operated patients whose decision on surgical candidacy had been solely based on MCTA. We observed that the operability decision had been uniquely posed on MCTA not only in a high proportion of patients with levels I and II disease, but also in up to half of the patients with level III disease. These results are consistent with previous studies, such as that conducted by Ley et al., in which MCTA provided the highest image quality and levels of sensitivity and specificity in segmental branches compared with the reference standard [14].

Furthermore, our approach attained significantly shorter time periods to fulfill complete the surgical assessment, which consequently resulted in reduced time lapse periods to surgery.

Additionally, pulmonary angiography is an expensive procedure, with total costs that range from 4084 to 6738 euros depending on patients’ severity profile [17]. Therefore, the constraint of this procedure to a restricted group of patients gains great relevance in national CTEPH referral centers, as it leads to significant optimization in terms of costs as well as in cardiac catheterization laboratory availability.

Focusing on safety concerns, the avoidance of PA in a considerable proportion of patients enabled to avert an invasive exam not exempted from complications. Prior large series reported a 1% rate of nonfatal major complications, 0.5% mortality rate, and 5% rate of minor complications [8,9]. This significantly differed from complication rates obtained in our series, which might be explained by the smaller sample size and additionally by growing expertise, more advanced catheters, and less aggressive vascular approaches in recent years.

The limitation of this study follows from its single-center design; thus, multicenter studies are needed with larger cohorts in order to evaluate the reproducibility of our results. It must be noted that high CT imaging interpretative expertise is required in order to assure an adequate operability assessment, and that our study did not aim to compare the validity of the two radiological methods but to show real-world practice. Moreover, as the radiological assessment is focused on offering the most suitable treatment to every patient in order to avoid denying surgery to operable patients; this requirement at initial assessment probably introduces bias and reinforces the level of intra and interobserver agreement.

## 5. Conclusions

On the basis of our findings, we support the idea that a noninvasive surgical assessment in patients diagnosed with CTEPH is feasible not only in patients with disease of levels I and II but also in a considerable proportion of patients with level III disease with no detrimental impact on postoperative outcomes. PA will still be necessary in approximately half of the patients with CTEPH in order to adequately determine surgical suitability as well as to guide and plan eventual balloon pulmonary angioplasty in non-operable patients. This approach leads to an evolutionary enhancement of contemporary measures of performance such as quality of care, availability gain, efficiency, and patient safety.

## Figures and Tables

**Table 1:** Intraoperative CTEPH classification of intervened patients regarding initial operability assessment.

Surgical CTEPH Classification	Decision-Based on MCTA *n* = 65	Complementary PA *n* = 26
Level I disease (%)	94.1	5.9
Level II disease (%)	75.6	24.4
Level III disease (%)	54.5	45.5

**Table 2:** Baseline and postoperative measures in operated patients.

Baseline and Postoperative Measures	Phase I (44)	Phase II (47)	*p*
Age (years)	56.74 ± 2.28	56.6 ± 1.76	ns
PAP mean prior PEA (mmHg)	46.87 ± 1.78	44.26 ± 2.01	ns
PVR prior PEA (UW)	7.55 ± 0.55	7.37 ± 0.64	ns
NT-proBNP (pg/mL)	1772 ± 428.34	1531.26 ± 291.97	ns
Pericardial effusion (*n*, %)	5 (11.36)	2 (4.2)	ns
6-MWD baseline (m)	508.81 ± 100.62	392.26 ± 20.93	ns
Surgical classification			
I (*n*, %) II (*n*, %) III (*n*, %)	9 (20.4) 20 (45.4) 15 (34.1)	9 (19.1) 23 (48.9) 15 (31.9)	ns
PAP m after PEA (mmHg)	32.17 ± 2.05	28.29 ± 2.52	ns
PVR after PEA (mmHg)	4.4 ± 0.54	3.78 ± 0.55	ns
Mean PAP m change (%)	−31.36	−36.08	ns
Mean PVR change (%)	−41.72	−48.71	ns
Residual PH after PEA *n* (%)	20 (46)	13 (27)	ns
Survival rate at 1 year after PEA	93.7%	95.4%	ns

PAP: pulmonary artery pressure; PEA: pulmonary endarterectomy; PVR: pulmonary vascular resistance; 6-MWD: 6-min walk distance; ns: not significant (*p* ≥ 0.001).
